# Auditory stimuli suppress contextual fear responses in safety learning independent of a possible safety meaning

**DOI:** 10.3389/fnbeh.2024.1415047

**Published:** 2024-10-10

**Authors:** Elena Mombelli, Denys Osypenko, Shriya Palchaudhuri, Christos Sourmpis, Johanni Brea, Olexiy Kochubey, Ralf Schneggenburger

**Affiliations:** ^1^Laboratory of Synaptic Mechanisms, Brain Mind Institute, School of Life Science, Ecole Polytechnique Fédérale de Lausanne (EPFL), Lausanne, Switzerland; ^2^Laboratory of Computational Neuroscience, Brain Mind Institute, School of Life Science and School of Computer and Communication Science, Ecole Polytechnique Fédérale de Lausanne (EPFL), Lausanne, Switzerland; ^3^Laboratory of Sensory Processing, Brain Mind Institute, School of Life Science, Ecole Polytechnique Fédérale de Lausanne, Lausanne, Switzerland

**Keywords:** safety learning, fear learning, freezing behavior, external inhibition, contextual fear memory, auditory-cued fear memory, startle response, valence

## Abstract

Safety learning allows the identification of non-threatening situations, a learning process instrumental for survival and psychic health. In contrast to fear learning, in which a sensory cue (conditioned stimulus, CS) is temporally linked to a mildly aversive stimulus (US), safety learning is studied by presenting the CS and US in an explicitly unpaired fashion. This leads to conditioned inhibition of fear responses, in which sensory cues can acquire a safety meaning (CS-). In one variant of safety learning, an auditory CS- was shown to reduce contextual fear responses during recall, as measured by freezing of mice. Here, we performed control experiments to test whether auditory stimuli might interfere with freezing by mechanisms other than safety learning, a phenomenon also called external inhibition. Surprisingly, when auditory stimulation was omitted during training (US-only controls), such stimuli still significantly suppressed contextual freezing during recall, indistinguishable from the reduction of freezing after regular safety training. The degree of this external inhibition was positively correlated with the levels of contextual freezing preceding the auditory stimulation. Correspondingly, in fear learning protocols which employ a new context during recall and therefore induce lower contextual freezing, auditory stimuli did not induce significant external inhibition. These experiments show that in safety learning protocols that employ contextual freezing, the freezing reduction caused by auditory stimuli during recall is dominated by external inhibition, rather than by learned safety. Thus, in safety learning experiments extensive controls should be performed to rule out possible intrinsic effects of sensory cues on freezing behavior.

## Introduction

In order to survive and adapt to an ever-changing environment, animals need to learn whether specific sensory cues are predictive for dangerous, or else for safe situations, and express the appropriate behavioral responses. The associative learning processes allowing such assignments are called fear−and safety learning (Blanchard and Blanchard, 1969; Rescorla, 1969; LeDoux, 2000; Maren, 2001; Christianson et al., 2012).

The introduction of simple and robust protocols of auditory-cued fear learning in rodents has facilitated the investigation of the neuronal circuits underlying fear learning (LeDoux, 2000; Maren, 2001; Tovote et al., 2015). In these aversive conditioning experiments, when an initially innocuous sensory cue, like a tone, is temporally paired with an aversive foot shock (the unconditioned stimulus, US), animals form an association between the tone and the US. Upon later presentation of the auditory stimulus alone, the animal will produce specific conditioned responses to the tone, which has therefore become a conditioned stimulus, CS+. In rodents, the conditioned responses are often measured in the form of behavioral immobility (“freezing”), but cardiovascular responses also occur (Blanchard and Blanchard, 1969; LeDoux et al., 1988; Fanselow, 2018).

Interestingly, early work has shown that using two CSs, one paired with the US, and the other one presented in an explicitly unpaired fashion with the US (CS+ and CS- [minus], respectively), the CS- can come to suppress the fear response evoked by the CS+ during recall. This phenomenon is called “conditioned inhibition”, and goes back to the work of Pavlov (Pavlov, 1927; Rescorla, 1969; Williams et al., 1992; Christianson et al., 2012; Sosa and Ramirez, 2019). Recent research has found that following explicitly unpaired training, the CS- furthermore comes to represent a “safety signal” for animals, with some evidence for a positive hedonic value of the CS- in mice (Rogan et al., 2005; Pollak et al., 2008). In these studies, suppression of the fear response by the CS- was tested by re−exposing the animals to the training context; the CS- was found to cause a *decrease* of contextual freezing (Rogan et al., 2005). Here, we will refer to this protocol as the “contextual freezing/CS- protocol” for safety learning. It is known, however, that certain sensory stimuli can suppress a given established conditioned response not as a consequence of a safety learning process, but rather, because of a direct interference of sensory stimulation with the conditioned response without prior learning, a phenomenon referred to as “external inhibition” (Pavlov, 1927; Myers and Davis, 2004; Christianson et al., 2012). Moreover, it has recently been shown that certain types of salient auditory stimuli like white noise, can cause flight-like movement responses of mice (Fadok et al., 2017; Hersman et al., 2020). Thus, the use of auditory cues as a CS- in safety experiments might be problematic if intrinsic properties of tone stimulation interfere with the freezing response of rodents.

The original aim of our work was to perform safety learning experiments with the contextual freezing/CS- protocol variant (Rogan et al., 2005; Pollak et al., 2008). In control experiments in which we omitted tone presentations during the training session we found, however, that auditory stimuli have an intrinsic propensity to suppress elevated levels of contextual freezing. In additional control experiments for auditory-cued fear learning, we found that auditory stimuli do not significantly influence residual levels of contextual freezing, most likely because the changed context in auditory-cued fear experiments induces smaller levels of contextual freezing. Taken together, our experiments suggest that the combination of tone stimuli, and elevated levels of contextual freezing can lead to misinterpretations in safety learning experiments, a finding which should be considered for future experiments in this domain.

## Materials and methods

### Animals

The behavioral experiments were performed using C57Bl/6J wildtype mice (*Mus musculus*), according to procedures authorized by the Veterinary office of the Canton of Vaud, Switzerland (authorizations VD 3518 and VD3518x1). To limit the variability of results due to sex differences in defensive behaviors (Maren et al., 1994; Pryce et al., 1999; Gruene et al., 2015), only male mice were used for the experiments. Six weeks old male C57Bl/6J mice were purchased from Charles River (France). The animals were kept group-housed under a 12/12 h light/dark cycle (lights on at 7 a.m.) for an initial 1–2 weeks, for acclimatization to the new environment. One week prior to behavioral testing, mice were transferred to single cages and habituated to handling by the experimenter for 5 min on each day, for five subsequent days. Handling and behavioral tests were conducted during the light phase. Mice had access to food and water ad libitum at all times. *N* = 48 C57Bl/6J mice were used for this study; no mice were excluded from the final datasets.

### Conditioning apparatus

Safety−and fear learning experiments were conducted in a conditioning apparatus (Video Fear Conditioning Optogenetics Package for Mouse, MED-VFC-OPTO-M, Med Associates Inc., Fairfax, VT, USA) under the control of VideoFreeze^®^ software (Med Associates Inc.). The habituation and training sessions of all experiments, as well as the recall session(s) of the safety learning protocols were performed within a red rectangular plexiglas enclosure (20 × 15 cm). This was placed on the metal grid floor of a conditioning chamber (VFC-008, Med Associates Inc.; grid floor connected to an ENV-414S stimulator), cleaned with 70% ethanol. This arrangement together will be referred to as “context A”. For the recall sessions in auditory-cued fear learning experiments and the related control experiments, the enclosure inside the conditioning chamber was formed by a white semi-circular plastic wall, the floor was a white smooth plastic surface, and the chamber was cleaned with perfumed general-purpose soap (context B).

The conditioning chamber was located in a sound-attenuated cubicle (NIR-022MD, Med Associates Inc.), equipped with a loudspeaker and a CMOS video camera (30 fps) with a near-infrared (IR) filter. All experiments were conducted under an array of white LEDs plus an array of IR LEDs.

### Behavioral protocols

Auditory stimuli (CS) were series of 100 ms tone beeps, repeated at 1 Hz for 30 s; each beep was a pure tone of 7 kHz auditory frequency at 80 dB with 5 ms rise time (2 ms for the initial experiments). Thus, 30 s long “tone blocks” or CSs resulted, which are indicated by light blue areas in the Figures.

The behavioral protocols used in short training protocols were as follows. On the first day, mice underwent an initial habituation session in context A, during which six tone blocks were applied at the times indicated in Table 1. One day later, a single training session was employed. For explicitly unpaired training sessions, the interval between each CS- and the subsequent US was variable (25–75 s; see Table 1), whereas the interval after each US and the following CS- was constant (60 s). For US only training sessions, six foot shocks were presented with the same timing as in the explicitly unpaired protocol (see Table 1). For paired CS+ / US training sessions (fear learning protocol), six CS+ / US pairs were given, with the CSs occurring at the times indicated in Table 1, and the foot shock starting at the end of the last tone beep. The foot shocks (1 s) had an intensity of 0.6 mA. For all the experiments with short training protocols, 1 day after the training session, a single recall session was employed, either in context A or context B, and six tone blocks (CS) were presented at the times indicated in Table 1.

**TABLE 1:** Timing of CS and US stimuli for 3-days behavioral protocols.

	Start time (s)	For US in explicitly unpaired: interval to preceding CS
CS1	120	
**US1**	204	55
CS2	265	
**US2**	329	35
CS3	390	
**US3**	494	75
CS4	555	
**US4**	629	45
CS5	690	
**US5**	744	25
CS6	805	
**US6**	899	65

The US times (left column) refer to explicitly unpaired protocols, and to US-only protocols. The right column indicates the time intervals between the end of each CS and the following US, in the explicitly unpaired training protocols.

For the safety learning experiment with prolonged training, a single habituation session (as above) was used, followed by two training sessions, and two recall sessions. In one additional US-only control, the habituation session contained no auditory stimuli (Supplementary Figure 1). The layout of the training sessions were of the explicitly unpaired CS-/US presentation type, or of the US-only type for controls, with small modifications compared to the other experiments. First, the number, and intensity of foot shocks was reduced to five, and to 0.4 mA, respectively. Furthermore, the variable timing intervals between each CS and its subsequent US as used in the short training protocols (Table 1), was slightly modified; the interval range was again 25 – 75 s.

All sessions had a duration of 1020 s, and the time intervals between sessions were 24 ± 1 h.

### Video recordings and analysis

The behavioral response of the mice was recorded at video rate (30 Hz) by the VideoFreeze^®^ software (Med Associates Inc.,). Based on the recorded videos, a movement index trace was generated using ezTrack software (Pennington et al., 2019). The movement index trace was used to compute a binary freezing trace using custom procedures in IgorPro 7 (WaveMetrics Inc., Lake Oswego, OR, USA). The animal was considered immobile (freezing state) if the movement index was below a threshold of 40 arbitrary units, for a minimum duration of 0.5 s. Traces of percent time spent freezing were calculated from the binary trace as a fraction of freezing state duration during each time bin (bin size, 10 s), and plotted as mean ± SEM for each experiment.

The freezing during the CS−and during the time windows preceding each CS was calculated from the binned trace as the average percent freezing during each 30s-long period (“CS” and “preCS” windows, respectively). For the CS−induced freezing difference, the baseline freezing during the preCS times was subtracted from the freezing during the CS periods. The data were calculated for each mouse, and plotted as mean ± SEM across animals for each experimental group.

### Correction for tone-driven startle responses

**FIGURE 1:**
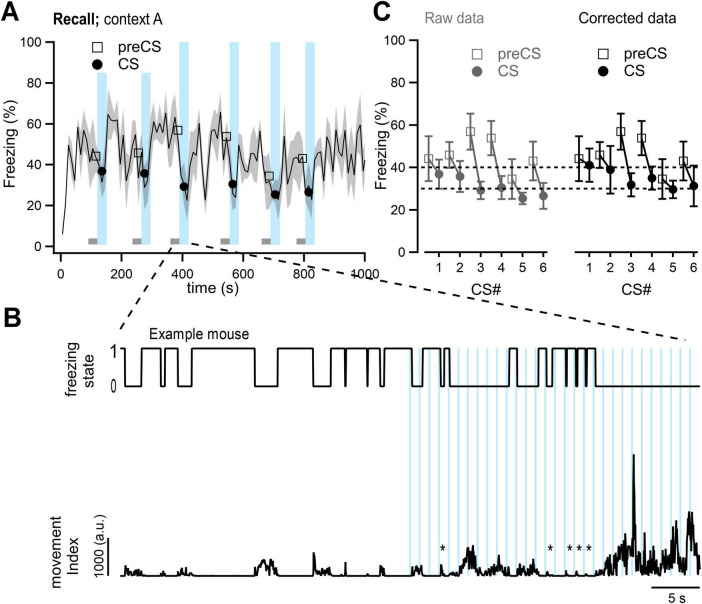
Occurrence of tone–evoked startle responses for some stimuli, and their correction. **(A)** The 10-s binned trace of percent time freezing estimate, averaged over *N* = 5 mice from the US-only control group of Figure 2 (same data as in Figure 2E, black traces). The 30 s blocks of auditory stimulation are shown by the light blue, vertical bars (see Materials and Methods for parameters of auditory stimulation). **(B)** A blow-up for an example mouse of the data set in panel **(A)** before and during the third tone block presentation. Top and bottom panels show the binary freezing state trace, and the movement trace resulting from the Videofreeze analysis, respectively. Stimulation with individual tone beeps (100 ms long, repeated at 1 Hz) are indicated by vertical light blue bars. Note that auditory stimulation led to an overall decrease in freezing. In addition, for some individual tone beeps, we observed head startle movements (see star symbols; see also Supplementary Video 1). *, indicates startle event. **(C)** Comparison of “percent time spent freezing” estimates before–(*left* panel) and after correction (*right*) for head startle. Freezing levels were calculated for the CS presentations (filled data points, “CS”) and for the 30 s periods immediately before each CS (open data points; “preCS”). The data during the CS underwent an additional head startle correction (see Materials and Methods). This led to a slight increase of the freezing estimate (compare black–and gray filled data points). The dashed horizontal lines indicate freezing levels of 30% and 40%.

In some mice exposed to tones in the training context, time-locked head movements in response to some of the individual tone beeps were observed (Figure 1B, star symbols; Supplementary Movie 1). These are likely startle responses evoked by the tones (Davis et al., 1993; Koch and Schnitzler, 1997). Since such responses were captured by the video-based analysis as movements (see also Pantoni et al., 2020), a slight underestimation of the “percent time spent freezing” estimate would result. To correct for this effect, we used a custom script in IgorPro 7 (WaveMetrics Inc., Lake Oswego, OR, USA). Transient movements initiated within 0.2 s from the onset of individual tone beeps, and with durations shorter than 0.9 s were detected, and blanked from the movement traces, so that each head startle occurrence was counted as freezing instead of movement. This correction was limited to the times of auditory stimulation during the training- and recall sessions (30-s CS periods), and led to a slight *increase* (a few percent) in the freezing estimate during CS presentation (see Figure 1C; compare black, and dark gray data points). In the Figures, averaged data points superimposed onto the 10-s binned freezing traces were *not* corrected for startle (see e.g. Figure 1A, filled data points). On the other hand, the freezing estimates during the CS presentations in all downstream analyses represent startle-corrected values (see e.g. filled data points in Figure 1C right.

### Statistical analysis

Statistical analysis was performed using GraphPad Prism 10 (GraphPad, San Diego, CA, USA). For the comparison between the freezing during the CS and preCS periods, as well as for the comparison of the CS -induced freezing difference between groups, we used a two-way repeated measures ANOVA (“two-way RM ANOVA” in the text). To test the significance of the CS−induced freezing difference averaged over all six CSs, we first performed Shapiro-Wilk normality tests on the individual data samples, which were all found to conform to a normal distribution. Significance was therefore tested with a one-sample t-test with zero as a hypothetical mean. The means between the groups were compared by a two-sample *t*-test (“*t*-test” in the text). The data are expressed as mean ± SEM. Statistical significance is indicated in the Figures using asterisks as *p* ≤ 0.05 (*), *p* ≤ 0.01 (**) and *p* ≤ 0.001 (***), or as “n.s.” for *p* > 0.05 (not significant).

## Results

### The safety learning protocol: Tone presentations reduce contextual freezing during recall

**FIGURE 2:**
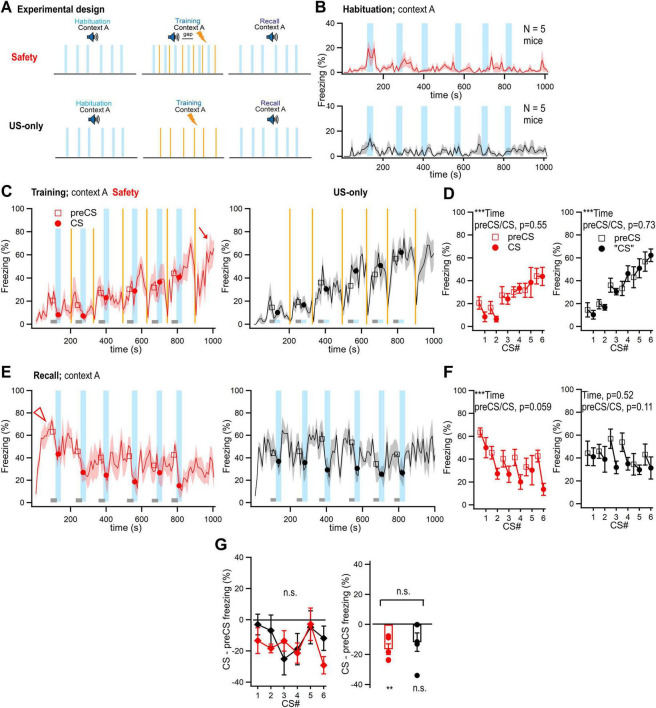
In a simple protocol for safety learning, US-only controls suggest that auditory stimuli cause external inhibition of contextual freezing. **(A)** Here, simple protocols of safety learning with a single training–and recall session were employed. One group of mice received a safety learning protocol with explicitly unpaired CS/US presentations (“Safety” group, *N* = 5 mice). A control group received only foot shocks during training, in an otherwise unchanged protocol (“US-only” group, *N* = 5 mice). **(B)** Time–resolved display of the average percent time spent freezing during the habituation session for the safety–and the US-only groups (red, and black data traces, respectively). **(C)** Traces of the average ± SEM percent time spent freezing during the training session for the Safety group (*left*, red data traces) and the US-only group (*right*, black data traces; *N* = 5 and 5 mice). Light vertical blue bars and orange lines indicate tone block (CS) presentation and foot shocks, respectively. The analysis times of freezing before each CS are indicated by the lower grey bars (30 s “preCS” epochs). The average time spent freezing (%) during each preCS and CS times are overlaid over the trace (open squares and filled circles). The data in this panel, and in panels B and E were not corrected for head startle. **(D)** Average time spent freezing (%) during the training sessions, for the CS periods and the preCS periods (closed, and open data points, respectively). Data for both the Safety–and the US-only group is shown (*left*, and *right* panel, respectively). The data in this panel, and in F and G were corrected for head startle. **(E)** Traces of average ± SEM percent time spent freezing during the recall session, for the Safety–and the US-only group (*left* and *right* panels, respectively; *N* = 5 and 5 mice). Lower grey boxes indicate the analysis window for preCS freezing. Note the partial suppression of freezing by tone stimuli in both groups (light vertical blue bars indicate time of auditory stimulation). **(F)** Average time spent freezing (%) during the CS periods for the recall session **(E)**, for both the Safety–and the US-only group (*left*, and *right* panel, respectively). In each case, freezing was analyzed during the pre-CS and CS periods (open, and closed data symbols, respectively). **(G)** *Left*, CS–induced *difference* in freezing for the six tone blocks during the recall session, averaged across all mice in each group (*N* = 5 and 5), for the Safety–and the US-only group (red, and black data, respectively). *Right*, the freezing difference values were averaged across all six tone blocks, but shown for the individual mice in each group. Note that there was no significant difference between the groups (*p* = 0.528; see “n.s.” symbol; *t*-test). For parameter values of further statistical comparisons, see Results. Error bars represent SEM. For parameter values for statistical tests, see Results. **p < 0.01; ***p < 0.001.

We started by implementing a previously described protocol for safety learning (Rogan et al., 2005; Figure 2A). During an initial habituation session, *n* = 6 tone blocks were applied (see Materials and Methods for details). The mice showed only little freezing in response to the tones during the habituation sessions (Figure 2B).

The essence of the safety learning protocol is to apply foot shocks (US) and tone blocks (CS-) with variable intervals (25−75 s, see Table 1 and Figures 2A, C; light blue areas and orange lines indicate tone blocks and foot shocks). These time intervals recapitulate the previously described “explicitly unpaired” presentations of tone blocks and foot shocks (Rogan et al., 2005). During such training sessions, the mice showed a gradual increase in freezing, interrupted by transient decreases in freezing following each foot shock. The decreases in freezing were caused by strong running−and jumping behavior of the mice during and after the foot shocks (Figure 2C, foot shocks marked by orange lines) (Fanselow, 1982). For quantification, we analyzed the average freezing during the 30 s tone blocks, and during 30 s baseline intervals preceding each tone block (“CS” and “preCS” periods, Figure 2C, vertical light blue lines and lower grey bars). The freezing estimate during the tone (CS) blocks was additionally corrected for startle-like head movements that were sometimes induced by tone beeps (see Material and Methods; Figure 1). A two-way RM ANOVA showed a significant effect of time (*p* < 0.0001), but not of preCs versus CS times (*p* = 0.552; Figure 2D, *left*). Thus, the freezing response of the mice gradually builds-up during the training session, and freezing during the tones is not different from the contextual freezing that precedes tone stimulation.

During the recall session one day later, mice were exposed to the same context, and tone blocks were presented in the absence of foot shocks (Figure 2E, *left*). Upon entering the conditioning chamber, mice developed maximal levels of contextual freezing within ∼ 100 s (Figure 2E, arrowhead). Presentation of tone blocks identical to the ones used for the habituation- and training sessions led to a decrease of freezing (Figure 2E, *left*; vertical light blue lines). Quantification of freezing during the tone blocks and 30-s pre-tone intervals, followed by a two-way RM ANOVA showed a significant effect of time (*p* < 0.0001), and a trend for the comparison between preCS−and CS times (preCS versus CS, *p* = 0.059; Figure 2F, *left*). The average CS- induced change in freezing across *N* = 5 mice in the group was -3% to -30% of the time spent freezing for each of the six tone blocks (Figure 2G, red data points). When averaging these values across all CSs for each mouse, a significant reduction of contextual freezing by the CS became apparent (*p* = 0.0094; one-sample *t*-test). This decrease in contextual freezing caused by the presentation of the CS- recapitulates previous observations with mice from safety learning protocols (Rogan et al., 2005; Pollak et al., 2008). Here, the observed reduction of freezing was not significant, maybe caused by the relatively small number of mice (*N* = 5), and by the relatively mild training regime.

### US-only control experiments suggest that external inhibition contributes to a reduction of freezing

It is known that phenomena of external inhibition, unrelated to learning processes, can contribute to the suppression of a learned conditioned response independent of safety learning (Pavlov, 1927; Myers and Davis, 2004; Christianson et al., 2012). To investigate whether external inhibition contributes to the reduction of contextual freezing in these experiments, we next carried out control experiments in which we omitted the tones during the training session (“US-only” controls; Figure 2A, *bottom*).

During the habituation session, freezing levels of the mice were low as expected (Figure 2B, black data traces). During the training session, mice showed a gradual increase in freezing, interrupted by decreased freezing after each foot shock, caused by running and jumping of the mice. To analyze the data quantitatively, we employed the same 30 s-time intervals as in the Safety group (Figure 2C, black trace; lower light blue and gray boxes). Freezing in these “fictive” CS and preCS intervals increased significantly with time (two-way RM ANOVA, *p* < 0.0001; Figure 2D, *right*), and, as expected, was not different between the fictive pre-CS versus CS times (two-way RM ANOVA, *p* = 0.728; Figure 2D, right).

For the recall session, mice were re-introduced to the same context, and standard tone blocks were applied. To our surprise, the tone blocks, although absent during the training session, appeared to reduce ongoing contextual freezing (Figure 2E, *right*, compare closed, and open average data points for tone and pre-tone intervals, *N* = 5 mice). The analysis of freezing during the recall session did not reveal an effect of time (two-way RM ANOVA; *p* = 0.517). There was a trend for a decreased freezing during auditory stimulation, which, however, did not reach significance (preCS versus CS, *p* = 0.113; two-way RM ANOVA, Figure 2F, *right*). The CS induced change in freezing showed that across tone blocks, auditory stimulation caused a reduction of freezing by -4% to -25% of the time spent freezing (Figure 2G, black data). Comparing this data to the corresponding one of the safety learning protocol revealed no significant difference (Figure 2G, *left*; *p* = 0.528, two-way RM ANOVA). Averaging the data for the US-only controls over all six tone blocks showed that the freezing decrease caused by auditory stimulation was not significant (*p* = 0.130, one-sample *t*-test; Figure 2G *right*, black data). Taken together, the US-only controls in Figure 2 suggest that during high fear states experienced by mice in the training context, exposing mice to a neutral (not previously conditioned) tone causes a suppression of contextual freezing. The average value of the suppression in the US-only group was not significantly different from the freezing reduction observed in the safety learning protocol (Figure 2G, right; *p* = 0.528).

### External inhibition of freezing by auditory stimulation in safety learning protocols with prolonged training

**FIGURE 3:**
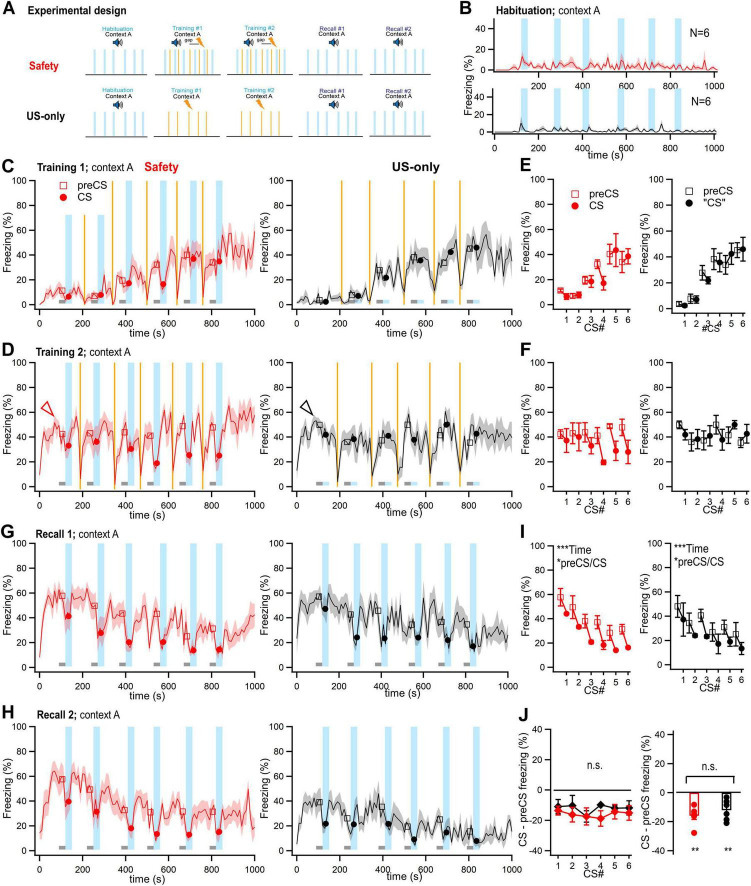
In prolonged safety learning protocols, auditory stimulation reduces fear responses irrespective of a possible safety meaning. **(A)** In these experiments, a prolonged training regime with two training sessions, and two recall sessions was employed. One group of mice received a regular safety training protocol with explicitly unpaired CS/US presentations (“Safety” group, *N* = 6 mice). A control group received only foot shocks during training, in an otherwise unchanged protocol (“US-only” group, N = 6 mice). **(B)** Time–resolved display of the average percent time spent freezing during the habituation session. **(C,D)** Traces of the average ± SEM percent time spent freezing during the first **(C)** and second training session **(D)**, for the Safety–and US-only group (N = 6 and 6 mice). Lines and symbols have the same meanings as in the corresponding panels of Figure 2. Note the elevated level of contextual freezing at the beginning of the second training session (arrowheads). The data in these panels, and in panels **(B,G,H)** were not corrected for head startle. **(E,F)** Average time spent freezing (%) during the preCS- and CS periods for the Safety group (*left* panels), and for the corresponding times for the US-only group (*right* panels). Data for the first and second training session are shown separately [(**E, F**) respectively]. The data in these panels, and in panels I and J were corrected for head startle. **(G,H)** Time–resolved average of the percent time spent freezing during the first **(G)** and second **(H)** recall sessions. Note that auditory stimulation (light blue bars) caused a suppression of freezing that appears similar in the Safety group (red traces, *left* panels) and in the US-only group (black traces, *right* panels). **(I)** Average time spent freezing (%) during the preCS- and CS periods for the Safety–and US-only group (*left*- and *right* panel, respectively). Note that both groups show a significant decrease in freezing between the preCS and CS times (see Results, for parameter values of statistical tests). **(J)** CS–induced change in freezing for the six tone blocks (*left* panel), and averaged across all tone blocks (*right* panel). The freezing change induced by auditory stimulation was not significantly different between the two groups (see Results for parameter values of statistical tests). Error bars represent SEM. For parameter values of statistical tests, see Results. *p < 0.05; **p < 0.01; ***p < 0.001.

In previous experiments with the contextual freezing/CS- protocol of safety learning, two training- and recall sessions have been employed (Rogan et al., 2005), whereas our initial experiments were based on single training- and recall sessions (Figure 2). Therefore, we next performed experiments in which we more closely mimicked the previous protocols (Rogan et al., 2005; see Materials and Methods for details). In these experiments, mice were assigned to two groups: A first group (*N* = 6) underwent explicitly unpaired presentations of tone blocks and foot shocks over two training sessions on two subsequent days (Figure 3A, *top*; “Safety learning” group). The second group underwent the same protocol, but no tone blocks were presented during the training sessions (Figure 3A, *bottom*; US-only group). Figures 3B–F show the behavior of the two groups of mice during the habituation, and training sessions. During the onset of the second training session, the mice showed a high level of freezing, indicative of a contextual fear memory acquired on the first day (Figure 3D, arrowheads; Fanselow, 1982, 2000). Inspection of the 10-s binned freezing traces suggested that during the recall sessions, mice in both the Safety learning−and control group showed a suppression of freezing by the tone blocks (Figures 3G–H; black and red traces, respectively; Supplementary Movies 2, 3). Quantitative analysis of freezing by two-way RM ANOVA showed a significant effect of time, and a significant difference between the preCS versus CS periods for both groups (Figure 3I; Safety learning group: Effect of time, *p* < 0.0001, preCS versus CS, *p* = 0.0159; US-only group: effect of time, *p* < 0.0001; preCs versus CS, *p* = 0.0345). Furthermore, the freezing difference induced by auditory stimulation was not significantly different between the two groups (*p* = 0.349, two-way RM ANOVA; Figure 3J, *left*). Averaging the values of CS-induced freezing difference for each group over all CS-presentations showed that the tone−induced decrease in freezing was significantly different from zero for both groups (Safety learning group, *p* = 0.0018; US-only group, *p* = 0.0093; one-sample t-test; Figure 3J, *right*). Furthermore, the values were not different between each other (*p* = 0.349, t-test; Figure 3J, *right*).

It might be argued that the exposure of mice to the auditory stimuli during the habituation session already leads to the formation of a safety memory, because the auditory stimulus occurred separate from the US (even if spaced by 24 h; see Figure 3A bottom; Materials and Methods). To exclude this possibility, we performed an additional US-only control experiment, in which auditory stimuli were also omitted during the habituation session (Supplementary Figure 1). The Results were similar to the US-only controls in Figure 3, that is, auditory stimulation presented for the first time during recall similarly caused a significant reduction of contextual freezing (effect of time, *p* < 0.0001; preCS versus CS, *p* = 0.0145, two-way RM ANOVA, Supplementary Figure 1I). This suggests that the reduction of contextual freezing by auditory stimuli during recall as tested in US-only control experiments (Figure 3G, black traces), is not caused by a process of safety learning across two sessions, but rather, represents a process of external inhibition (Pavlov, 1927; Myers and Davis, 2004; Christianson et al., 2012). Thus, care has to be taken when experiments with safety learning protocols are performed, and appropriate controls addressing possible effects of external inhibition should be performed.

### Suppression of freezing by intrinsic properties of tone stimulation is not significant in fear learning experiments

**FIGURE 4:**
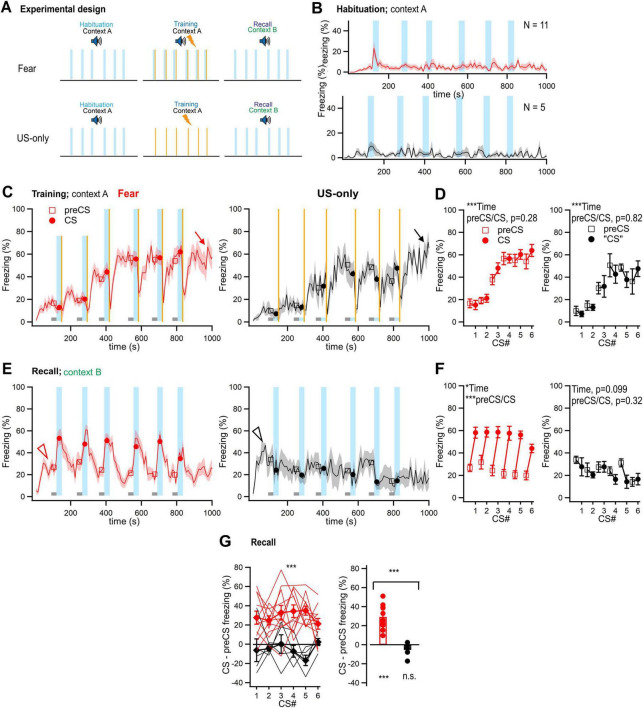
No significant suppression of freezing by external inhibition in fear learning experiments. **(A)** Scheme of the fear learning protocol, and of the US-only controls. Note that a novel context (context B) was used for the recall session in both groups. *N* = 11 and 5 mice were investigated in the fear learning–and the US-only group, respectively. **(B)** Time–resolved average percent time spent freezing for the habituation session of each group. **(C)** Time–resolved display of the average percent time spent freezing for both groups. Lines and symbols have the same meanings as in the corresponding panels of Figures 2, 3. The arrows mark the ongoing contextual freezing long after the last CS/US presentations (or after the last US for the US-only group). The data in this panel, and in panels **(B,E)** were not corrected for head startle. **(D)** Average percent time spent freezing during the preCS and CS periods of the training session, for both groups. The data in this panel, and in **(F,G)** were corrected for head startle. **(E)** Time–resolved average trace of the percent time spent freezing during the recall sessions for both groups. Note some level of “residual” context freezing (arrowheads) despite the changed context employed for the recall sessions. **(F)** Average percent time spent freezing during the preCS- and CS periods for the Fear learning and the US-only group (left - and right panel, respectively). Tone stimulation significantly increased freezing in the fear learning group as expected (red data, left panel), but not in the US-only group (right). See Results for parameter values of statistical tests. **(G)** CS–induced change in freezing for the six tone blocks (left panel), and averaged across all tone blocks (right panel), for both groups. The freezing change induced by tone blocks was significantly different between the two groups (see Results for parameter values of statistical tests). Error bars represent SEM. For parameter values of statistical tests, see Results. *p < 0.05; ***p < 0.001.

In auditory-cued fear learning experiments, the paired presentation of a CS with a US (thus, CS+), leads to a CS driven *increase* in freezing in a recall session (see LeDoux, 2000; Tovote et al., 2015; Palchaudhuri et al., 2022 for reviews). In such experiments, the general properties of the conditioning chamber, such as floor materials, wall shape, and olfactory cues (together called “context”) are changed for the recall session, in order to unmask CS driven freezing from the contextual fear response that would otherwise prevail. Nevertheless, a certain amount of residual context freezing remains in these conditions (see below). Because we found that auditory stimuli readily act as an external inhibitor to reduce elevated levels of contextual freezing, an *underestimation* of CS+ driven freezing would result, if external inhibition was present also in fear learning experiments. To test this possibility, we next performed fear learning experiments with paired CS/US presentations (“Fear” group), or with foot shocks alone on the training day (“US-only” control group). Both groups experienced tone blocks in a new context during a recall session (Figure 4A). During the training session, mice showed gradually increasing freezing levels, with freezing that continued after the last foot shock in both groups (Figure 4C, arrows), and with a significant effect of time, but not of pre-CS versus CS periods (Figure 4D; two-way RM ANOVA; for the Fear group: time, *p* < 0.0001; preCS versus CS, *p* = 0.284; for the US-only group: time, *p* < 0.0001; preCS versus CS, *p* = 0.816). Thus, the freezing response during the training session largely reflects a build-up of contextual freezing, and tone stimuli (CS) do not seem to have yet acquired an aversive meaning for the mice (see Discussion).

During the recall session, the mice in both groups showed a moderate, initial increase in contextual freezing (Figure 4E, arrowheads; residual context freezing, see above). Presentation of tone blocks led to significant increases in freezing in the fear learning group, which shows that auditory stimuli have acquired the meaning of an aversively−motivated CS+ (Figures 4E, F; red data symbols; *p* < 0.0001 for preCS versus CS times; two-way RM ANOVA). On the other hand, in the US-only controls, tone applications had no effect on the residual context freezing (*p* = 0.318 for preCS versus CS; two-way RM ANOVA; Figures 4E, F; black data symbols). The tone−induced change in freezing was significantly different between the two groups (*p* < 0.0001, two-way RM ANOVA; Figure 4G *left*). Averaging the values of CS-induced freezing difference for each group over the six tone blocks revealed a significant difference from zero for the Fear group (*p* < 0.0001; one-sample *t*-test), but not for the US-only group (*p* = 0.186; one-sample t-test; Figure 4G, *right*). Furthermore, the freezing difference induced by auditory stimulation was significantly different between the two groups (*p* = 0.0004, *t*-test; Figure 4G, *right*). We conclude that the influence of external inhibition on ongoing freezing behavior is not significant in fear learning experiments. The reason is likely that in auditory-cued fear learning a changed context is employed for the recall session, and therefore, contextual freezing is lower than in the contextual freezing/CS- protocol of safety learning.

### The amount of external inhibition correlates with context freezing

**FIGURE 5:**
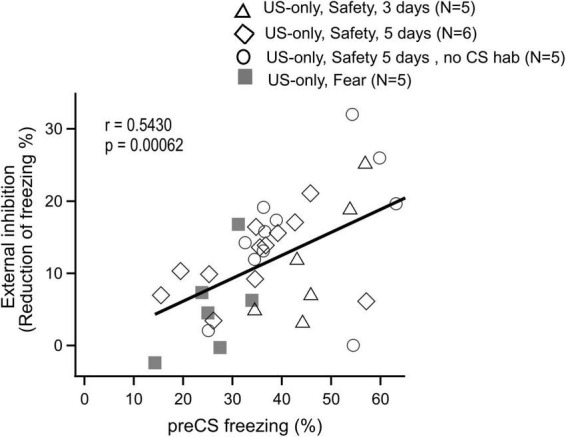
The amount of external inhibition correlates with the level of contextual freezing immediately before auditory stimulus presentation. The amount of external inhibition during the recall sessions as assessed in US-only control experiments (i.e. the tone-induced *reduction* of freezing), was plotted as a function of freezing occurring immediately before auditory stimulation (preCS periods). Data from all US-only conditions were included: Figure 2E *right*; Figures 3G, H, *right*; Supplementary Figure 1G and Figure 4E, right (indicated by labels, “US-only, Safety, 3 days”; “US-only, Safety, 5 days”; “US-Only, Safety 5days, no CS hab”; and “US-only, Fear”, respectively). Each data point is the average across all mice in the respective groups (N = 5 or 6 as indicated). The data indicates a correlation between the two measures (*r* = 0.543; *p* = 0.00062).

To provide quantitative support for the latter conclusion, we plotted the degree of external inhibition as a function of pre-CS freezing, extracting data from all US-only control experiments. This revealed a positive correlation between the amount of external inhibition caused by auditory stimulation, and the immediately preceding contextual freezing (Figure 5; *r* = 0.543; *p* = 0.00062). This strongly suggests that the degree of external inhibition depends on the amount of contextual freezing that precedes auditory stimulation.

### No ambiguity of unpaired controls for auditory-cued fear learning by a possible contribution of safety learning

**FIGURE 6:**
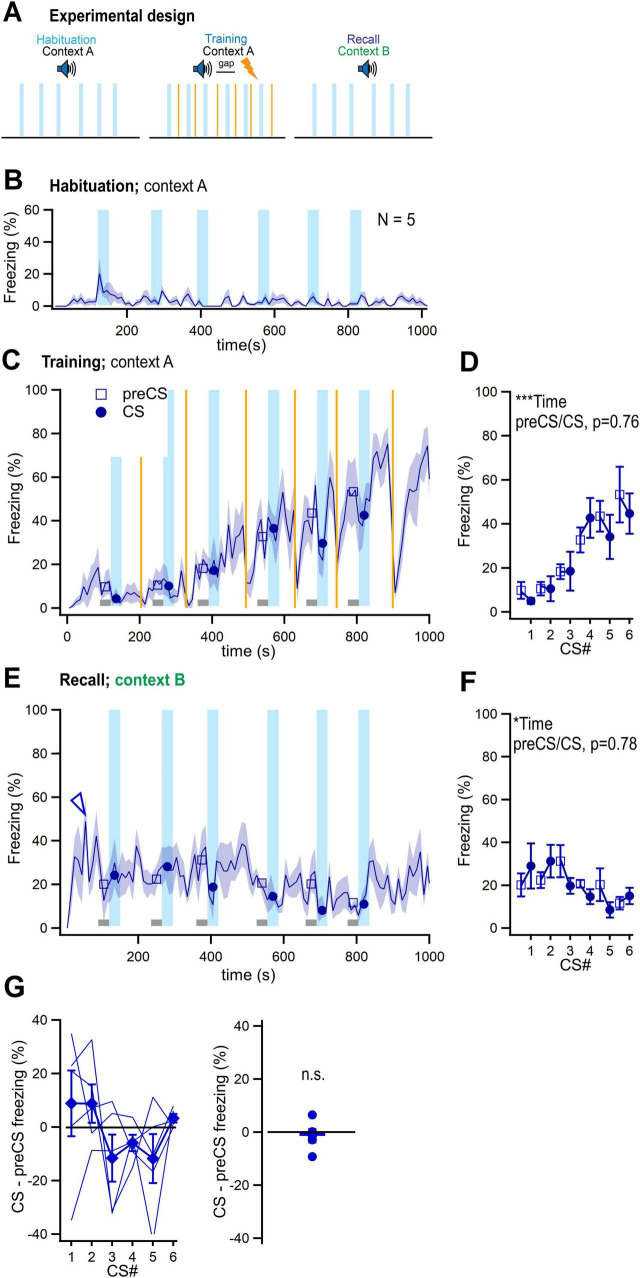
An “unpaired” control experiment for fear learning is neither influenced by a possible safety association, nor by intrinsic suppression of freezing by tones. **(A)** Scheme of the experimental protocol, with explicitly unpaired presentation of tones and foot shocks during the training session, and changed context during the recall session. **(B)** Time–resolved display of the average percent time spent freezing during the habituation session. Group size, *N* = 5 mice. **(C)** Time–resolved display of the average percent time spent freezing during the training session. Lines and symbols have the same meaning as in the corresponding panels of Figures 1, 2–4. The data in this panel and in panels **(B,E)** were not corrected for head startle. **(D)** Average percent time spent freezing during the preCS and CS periods of the training session. The data in this panel, and in **(F,G)** were corrected for head startle. **(E)** Time–resolved average ± S.E.M. of the percent time spent freezing during the recall session. **(F)** Average percent time spent freezing during the preCS- and CS periods. Note that tone stimulation did not significantly change freezing. See Results for parameter values of statistical tests. **(G)** CS–induced change in freezing for the six tone blocks (left panel), and averaged across all tone blocks (right panel). The tone blocks did not induce a significant change in freezing (see Results for parameter values of statistical tests). Error bars represent SEM. For parameter values of statistical tests, see Results. *P < 0.05; ***p < 0.001.

In experiments with auditory-cued fear learning protocols, “unpaired” CS and US presentations are often used as controls to remove temporal association between the CS and the US, while applying the same number of stimuli (see e.g. Rumpel et al., 2005; Clem and Huganir, 2010). As was already pointed out by Rescorla, in case such controls are set-up in a way that a *negative* contingency between CS and US results (i.e. the US will *never* occur during the CS), then one expects that tone stimulations acquire a safety meaning, at least for prolonged training conditions (Rescorla, 1967). For the purpose of a control experiment for fear learning, such an effect is unwanted, because any *ex-vivo* changes of synaptic properties measured in such experiments (Rumpel et al., 2005; Clem and Huganir, 2010) could then result−in the control group−from safety learning. To address this possibility, we performed a further control experiment. We presented explicitly unpaired CS / US stimuli during a training session, as one would do in protocols of safety learning or during “unpaired” control experiments for fear learning. The effects of auditory stimuli were then tested in the *novel* context B that is typical for auditory-cued fear learning experiments (Figure 6A).

The freezing behavior of mice during the habituation session, and the training session were similar to the behavior observed in the safety learning protocols (Figures 6B–D, *N* = 5 mice; compare with Figure 2C). During recall in the novel context B, residual contextual freezing was observed (Figure 6E, arrowhead). Presentation of tone blocks led to only small, and variable effects on this residual contextual freezing (Figures 6E, F; two-way RM ANOVA, *p* = 0.780 for preCS versus CS). Correspondingly, the CS-induced changes in freezing levels were not significantly different from zero (Figure 6G; *p* = 0.617, one-sample *t*-test).

These experiments show that unpaired controls in fear learning experiments are not strongly influenced by unwanted processes of safety learning, at least with the mild training conditions employed in Figure 6 typical for fear learning experiments. Nevertheless, a negative contingency between US and CS as present in our experiments of Figure 6, should best be avoided in the design of unpaired controls for fear learning experiments (Rescorla, 1967).

## Discussion

The concept of conditioned inhibition, or safety learning in Pavlovian conditioning experiments goes back to the seminal work by Pavlov (Pavlov, 1927; Rescorla, 1969; Williams et al., 1992). Recent research has shown that auditory stimuli applied in an explicitly unpaired fashion with foot shock stimulation (US), come to suppress contextual freezing in mice during a recall session. Furthermore, such sensory cues might acquire a positive hedonic value, for example by suppressing depressive states in the mouse model (Rogan et al., 2005; Pollak et al., 2008). In many behavioral protocols with aversively motivated conditioning and with safety learning, freezing of mice or rats is used as a read-out of the fear state of the animal (Blanchard and Blanchard, 1969; Fanselow, 1982; Bourtchuladze et al., 1994; LeDoux, 2000; Rogan et al., 2005; Tovote et al., 2015). Here, we performed a series of control experiments for auditory-cued safety- and fear learning experiments, to test whether auditory stimuli *per se* can interact with the freezing behavior of mice. We analyzed freezing in a time-resolved manner, which allowed us to easily visualize changes in freezing. We found, surprisingly, that tone stimulation with similar properties as standardly used in auditory-cued fear learning experiments, has an intrinsic propensity to suppress elevated contextual freezing in safety learning experiments, and thus, causes external inhibition (Figures 2, 3). This effect is not significant in auditory-cued fear learning experiments, because in the latter, the use of a changed context during recall leads to smaller levels of contextual freezing (Figure 4). Indeed, we found that the strength of external inhibition correlates with the level of context freezing, when the data was analyzed across all US-only control experiments (Figure 5). We conclude that care has to be taken when designing safety learning protocols. Extensive control experiments should be performed to validate whether a given sensory cue has an intrinsic propensity to interrupt freezing.

We have investigated here a safety learning protocol of the contextual freezing/CS- variant, in which an auditory stimulus is presented explicitly unpaired with the US during training; the auditory stimulus is then presented again in the same context in a recall session (Rogan et al., 2005; Pollak et al., 2008). Of note, there are other types of safety learning protocols, in which two CSs of different sensory modalities are used during training, one paired (CS+), and the other one explicitly unpaired with the US (CS-). Compound presentation of the CS+ and the CS− during recall, over several days, then induce lower freezing responses as compared to the CS+ alone (e.g. Foilb et al., 2016). In addition, such “CS+/CS- compound” protocols have also been used in combination with fear-potentiated startle as a read-out of fear and conditioned inhibition (Falls and Davis, 1995; Heldt and Falls, 2003). In these implementations of safety learning, controls for external inhibition have been applied (Myers and Davis, 2004; Foilb et al., 2016; Yau and McNally, 2022).

We found that brief auditory “beeps” (100 ms) used for auditory CSs in standard fear learning protocols, can cause time−locked head movements in some mice. These were especially apparent when freezing levels were high, i.e. during the recall session of safety learning protocols and their US-only controls, when the training context was employed (Figures 2, 3). We interpret the tone-beep driven movements as startle-like responses. The startle response is an unconditional reflex that can occur after sharp-onset, high amplitude unexpected tones; startle responses in rodents are well-known to be potentiated by fear learning (Davis et al., 1982; Davis et al., 1993; Koch and Schnitzler, 1997). In our experiments, startle-like responses were most obvious for the head and neck regions of the mice (Supplementary Video 1). These startle responses with a duration of a few 100 ms were picked-up by the video-based analysis (Figure 1; Pantoni et al., 2020) and caused an apparent reduction in the freezing estimate. Because the mice stayed otherwise immobile, we assume that without the startle response, mice would be in the freezing state, and corrected the freezing estimate during the CS periods accordingly. Thus, startle-like movements evoked by tone stimulation can further aggravate the tone−evoked suppression of freezing in safety learning experiments (Figure 1). Note, however, that tone-evoked startle responses accounted for only a small fraction of external inhibition uncovered here. Future work with auditory cued fear- and safety learning protocols should take the presence of tone-evoked startle-responses into account, possibly correcting for startle-like effects in the analysis of freezing behavior.

Our finding that stimulation with pure tones can lead to a suppression of freezing, especially in a high-fear state of mice driven by contextual cues, bears a relation to recent studies investigating the effect of white noise auditory stimulation on escape behavior in mice (Fadok et al., 2017; Hersman et al., 2020; Totty et al., 2021; Trott et al., 2022). It was shown that an escape behavior induced by white noise depended on the fear state of mice, being observed more faithfully in aversively-motivated conditioning contexts (Fadok et al., 2017; Trott et al., 2022). Recent data suggest that the flight responses induced by white noise might represent, at least in part, an unconditional response driven by a high saliency of these types of auditory stimuli (Hersman et al., 2020). Moreover, one study showed that an unconditional movement response can be driven by previously non-paired auditory pure tone stimulation, although the pure tones caused a less vigorous movement response than the white noise stimuli (see Figure 5 of Trott et al., 2022). In our case, the reduction of freezing of mice in a high-fear state by auditory pure tone stimulation is an analogous behavioral phenomenon of movement initiation, although the movement triggered in our experiments (performed in small conditioning chambers), was less vigorous than an escape reaction, and often consisted of limited head−and upper body movements (see Supplementary Movies). Nevertheless, our finding of external inhibition indicates that even the smaller salience of an auditory pure tone stimulus was sufficient to trigger movement initiation, therefore bearing some similarity with the recent white noise stimulation studies (Hersman et al., 2020; Trott et al., 2022). Together, these findings illustrate that the saliency of auditory stimuli, and their interaction with the freezing- and movement behavior of rodents, can interfere with conclusions derived from analyzing ongoing freezing behavior in safety learning experiments. In addition, it remains possible that more cognitive parameters of the stimulus, like its “novelty”, contribute to external inhibition, because we observed external inhibition also when we omitted the auditory stimuli from all sessions prior to recall (Supplementary Figure 1). However, regardless of whether external inhibition is caused by the novelty of the auditory stimuli, and/or by a more aversive connotation of auditory stimulation in the sensory-motor regime, it is clear that the propensity of sound stimuli in producing external inhibition needs to be taken into account in fear- and safety learning experiments.

In the course of the experiments presented here, we have used three CS/US contingencies during the training day, i.e. CS-/US explicitly unpaired conditions, US-only, and CS+/US pairing. We find that the freezing behavior on the (first) training day is similar between the three conditions, and follows a gradual increase in freezing, which roughly tracks the cumulative number of past foot shock stimuli (see panels C of Figures 2–4, 6). We found that tone stimulation did not have a significant effect on freezing during the training sessions (this applies to the *first* training sessions in case of the prolonged training regimes; Figure 3), neither with paired CS/US contingencies, nor in explicitly unpaired conditions. It is sometimes assumed that the time−dependent increase in freezing during the training session of auditory-cued fear experiments, when implicitly analyzed *only* for the CS-times, would indicate an ongoing acquisition of a cued fear memory. Nevertheless, the experiments performed here under various CS/US contingencies show that freezing during training is mainly determined by an ongoing process of contextual fear learning (Fanselow, 2000). Thus, the effect of a CS+ in evoking freezing as a conditioned response in fear learning experiments might be observable for the first time only during fear memory recall, when cued memory is tested in a different context (e.g. Figures 4E, F; red data symbols). It is possible that time-dependent processes of memory consolidation need to take place, before the CS can evoke a conditioned response (Bourtchuladze et al., 1994). An alternative explanation would be that the absence of tone-evoked changes in freezing on the training day, is caused by a masking of tone−evoked freezing by already elevated levels of contextual freezing, but we regard this as an unlikely explanation.

We have obtained evidence that external inhibition dominates the reduction of contextual freezing induced by tone stimulation in a contextual freezing/CS- protocol of safety learning. Nevertheless, we do not intend to conclude that there were no processes of safety learning in our experiments. For example, during safety learning protocols with two training sessions, auditory stimuli started to visibly suppress freezing in the second training session from roughly the fourth stimulation onwards (see Figures 3D, F; red data). It is possible that this phenomenon, which we did not investigate further, represents the start of a safety assignment to the auditory stimulus, which might only be observable after a certain degree of training. Nevertheless, the tone-induced suppression of contextual freezing during the recall session (Figures 3G–J, red data symbols) was similarly observed when no auditory stimuli were present during the training session (Figures 3G–J, black data, and Supplementary Figure 1), indicating that the latter response is largely driven by external inhibition.

In summary, we have performed a series of interrelated control experiments for auditory-cued safety, and fear learning experiments. The results show that in safety learning protocols of the contextual freezing/CS- variant, auditory stimulation has an intrinsic propensity to suppress elevated levels of freezing, and thus, cause external inhibition. This effect was not significant in auditory-cued fear learning experiments, in which levels of contextual freezing are lower. The data show that care must be taken in future safety learning experiments that aim to use distinct auditory cue as a “safety signal”, especially when tested in the background of elevated levels of contextual freezing.

## Data Availability

The raw data of this study are accessible at Zenodo (doi: 10.5281/zenodo.13524007).

## Supplementary material

Supplementary Figure 1 **(A)** Here, an additional variation of the US-only control experiment for safety learning was performed, in which no auditory stimuli were given during the habituation session. **(B–J)** The layout of the panels is the same as in Figures 3B–J. Note that despite the absence of auditory stimuli in both the habituation−and training sessions, auditory stimulation leads to a significant decrease of contextual freezing during the recall session (panel **G,I**; Effect of Time, *p* < 0.0001; preCs versus CS, *p* = 0.0145; two-way RM ANOVA). Furthermore, the freezing difference is significantly different from zero (*p* = 0.0252; one-sample t-test; panel **J**, right).

Supplementary Movie 1 The movie shows an example mouse of the US-only group in Figure 2, for a 30-s interval before, and after the onset of auditory stimulation. In the animation above the video, the binary freezing state trace, and the movement trace is shown, as well as timing of individual 100 ms tone beeps (vertical light blue lines). This mouse is the same as the one illustrated in Figure 1B.

Supplementary Movie 2 Video of an example mouse in the Safety group of Figure 3, during the second auditory stimulation (CS) of the first recall session. Note that this mouse showed relatively low levels of freezing even before the CS presentation.

Supplementary Movie 3 Video of an example mouse in the US-only group of Figure 3, during the second auditory stimulation (CS) of the first recall session. This mouse shows a marked movement onset upon CS presentation.
