# Supplementary figures and images for: Auditory stimuli suppress contextual fear responses in safety learning independent of a possible safety meaning
Source: Front Behav Neurosci. 2024 Oct 10;18:1415047. doi: 10.3389/fnbeh.2024.1415047 (PMC11499156; doi:10.3389/fnbeh.2024.1415047)

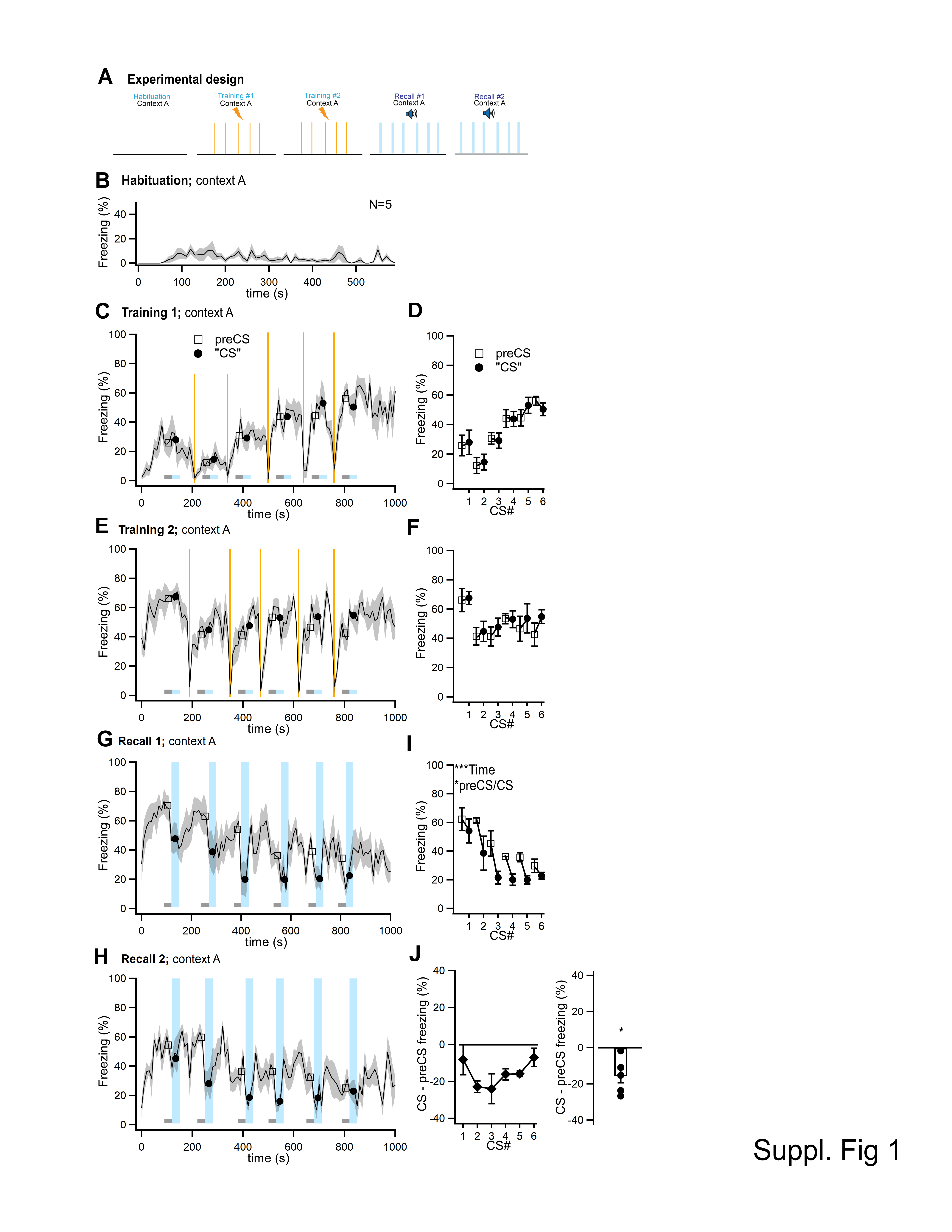

Supplement: Supplementary Figure 1 — (A) Here, an additional variation of the US-only control experiment for safety learning was performed, in which no auditory stimuli were given during the habituation session. (B–J) The layout of the panels is the same as in Figures 3B–J. Note that despite the absence of auditory stimuli in both the habituation−and training sessions, auditory stimulation leads to a significant decrease of contextual freezing during the recall session (panel G,I; Effect of Time, p < 0.0001; preCs versus CS, p = 0.0145; two-way RM ANOVA). Furthermore, the freezing difference is significantly different from zero (p = 0.0252; one-sample t-test; panel J, right). [file Image_1.TIF]
